# Comparison of the Predictive Capacity of Oxygenation Parameters, Oxygenation Indices, and CURB-65 to Mortality, Mechanical Ventilation, and Vasopressor Support in Community-Acquired Pneumonia at Different Altitudes

**DOI:** 10.1155/carj/9378618

**Published:** 2025-03-11

**Authors:** Eduardo Tuta-Quintero, Alirio R. Bastidas, Angelica Mora, Luis F. Reyes, Laura E. Bello, Alejandra P. Nonzoque, Laura D. Saza, Natalia Trujillo, Jenifer C. Arias, Paola Mejía Martinez, Daniel Osorio, Paola Narváez, Laura Perdomo, Luis Vargas, María Pérez, Jesus Rubiano, Paula Pinillos, Juan Naranjo, Angela María Martínez

**Affiliations:** ^1^Department of Internal Medicine, Universidad de La Sabana, Chía, Colombia; ^2^Unisabana Center for Translational Science, School of Medicine, Universidad de La Sabana, Chia, Colombia

**Keywords:** altitude, artificial, intensive care units, pneumonia, respiration, vasoconstrictor agents

## Abstract

**Background:** Populations residing at high altitudes display distinct physiological adaptations that are essential for understanding respiratory diseases. However, there is limited research on how these adaptations affect the assessment and prognosis of community-acquired pneumonia (CAP).

**Methods:** A prognostic validation nested within a retrospective cohort was conducted on subjects with pneumonia admitted to two high-complexity institutions in Colombia at different altitudes above sea level. The receiver operating characteristic (ROC) curves were calculated for SaO_2_, PaO_2_, SpO_2_, A-a O_2_ gradient, a-A index, PaO_2_/FiO_2_, SpO_2_/FiO_2_, and the CURB-65 score to predict 30-day mortality, requirement for invasive mechanical ventilation (IMV), and need for vasopressor support.

**Results:** 3467 were selected for analysis, with 73.7% (2557/3467) residing at high altitudes and 26.2% (910/3467) at low altitudes. The CURB-65 score  ≥ 2 showed a performance in predicting mortality of 0.707 (95% CI: 0.653–0.761; *p* < 0.001) at low altitudes and 0.737 (95% CI: 0.709–0.765; *p* < 0.001) at high altitudes. The PaO_2_/FiO_2_ ≤ 300 showed a performance in predicting the need for IMV and vasopressor support of 0.734 (95% CI: 0.685–0.783; *p* < 0.001) and 0.724 (0.674–0.775; *p* < 0.001) at high altitudes, respectively. The SpO_2_/FiO_2_ ≤ 350 showed a performance in predicting the need for IMV of 0.679 (0.507–0.85; *p* < 0.001) at low altitudes. The A-a O_2_ gradient ≥ 10 showed a performance in predicting the need for vasopressor support of 0.686 (95% CI: 0.537–0.835; *p*=0.06) at low altitudes.

**Conclusion:** In patients with CAP at altitudes above 2500 m above sea level, PaO_2_/FiO_2_, SpO_2_/FiO_2_, and the A-a O_2_ gradient show a greater predictive capacity for 30-day mortality, need for IMV, and vasopressor requirements. The CURB-65 score showed a good predictive performance.

## 1. Introduction

Community-acquired pneumonia (CAP) is a prevalent acute respiratory infection [1, 2]. In North America, its annual incidence is reported at 24.8 cases per 10,000 adults [1, 2]. Among hospitalized populations, this incidence rises significantly to 649 cases per 100,000 adults, with an average hospitalization rate of 464.8 per 100,000 individuals [2]. Mortality rates associated with CAP are notable, reaching 13% at 30 days, 23.4% at 6 months, and 30.6% at 1 year [1, 2]. Mortality risk has been linked to factors such as advanced age, comorbidities, the extent of multiorgan involvement, and low blood oxygenation levels [3, 4]. Notably, individuals residing at high altitudes who develop CAP may experience more severe hypoxemia, which could influence their clinical course and affect the accuracy of prognostic scoring systems used to predict outcomes [4–6].

Populations residing at high altitudes (above 2500 m) exhibit distinct physiological adaptations that have been widely studied in the context of respiratory diseases [7–9]. These physiological adaptations include increased lung capacity driven by chronic hyperventilation, enhanced oxygen uptake, and greater vital capacity [10–12]. Hematological changes include elevated red blood cell counts stimulated by erythropoietin, increased capillary density in tissues, and higher hemoglobin concentrations, all of which enhance oxygen transport efficiency [13–15].

Various scales are used to evaluate a patient's oxygenation status, such as blood oxygen saturation (SaO_2_), partial pressure of oxygen (PaO_2_), peripheral capillary oxygen saturation (SpO_2_), alveolar–arterial oxygen gradient (A-a O_2_ gradient), alveolar–arterial oxygen index (a-A index), the arterial oxygen pressure to inspired oxygen fraction ratio (PaO_2_/FiO_2_), and peripheral oxygen saturation to fraction of inspired oxygen index (SpO_2_/FiO_2_) [10–17]. However, further research is needed to explore altitude-related differences in oxygenation between healthy individuals and patients to improve the accurate assessment of respiratory infectious disease severity and prognosis in high-altitude environments [16–18].

Considering and evaluating the values of oxygenation parameters (SaO_2_, PaO_2_, and SpO_2_) and indices (A-a O_2_ gradient, a-A index, PaO_2_/FiO_2_, and SpO_2_/FiO_2_), as well as their variations with altitude, in clinical practice could provide a more accurate understanding of clinical status and improve predictions of ventilatory support needs and mortality in patients with CAP [7]. However, data on its values in infectious diseases remain limited in the scientific literature [19, 20]. The aim of this study was to evaluate and compare the performance of SaO_2_, PaO_2_, SpO_2_, A-a O_2_ gradient, a-A index, PaO_2_/FiO_2_, SpO_2_/FiO_2_, and the CURB-65 score for clinical outcomes in patients residing at high versus low altitudes with a diagnosis of CAP.

## 2. Methods

A prognostic validation was nested within a multicentre retrospective cohort study in patients with CAP admitted to two hospitals; one located at high altitudes (> 2500 m above sea level) and the other at low altitudes (< 2500 m above sea level). The review period for medical records was from January 2010 to December 2020, covering 10 years, and data were exclusively extracted from the medical records of hospitalized patients during that time.

### 2.1. Outcomes

The main objective of the study was to assess the predictive capacity of oxygenation parameters, oxygenation indices, and the CURB-65 score regarding mortality at 30 days, the need for invasive mechanical ventilation (IMV), and the requirement for vasopressor support at high and low altitudes in patients hospitalized for CAP.

### 2.2. Eligibility Criteria

Participants of both sexes, over 18 years of age, with a diagnosis of CAP admitted to the emergency department or intensive care unit (ICU) for this same pathology were recruited. The diagnosis of CAP was established following the 2007 ATS/IDSA criteria [21], which were reviewed in 2019 [22]. These criteria include the presence of specific clinical features such as cough, fever, sputum production, and pleuritic chest pain, typically supported by lung imaging, mainly chest radiographs [21, 23]. Regarding oxygenation indices and parameters, measurements were made based on established parameters to assess the severity of hypoxemia and respiratory failure in patients with CAP, including: PaO_2_ ≤ 60 mmHg, SaO_2_ ≤ 90%, A-a O_2_ gradient ≥ 10, a/A ratio ≤ 0.8, PaO_2_/FiO_2_ ≤ 300, SpO_2_ ≤ 90%, and SpO_2_/FiO_2_ ≤ 350 [24–26]. Patients with nosocomial infection, incomplete clinical history, and absence of information on 30-day survival were excluded.

The population was divided into two groups based on their altitude of residence: low altitudes and high altitudes. A minimum residence duration of 1 year at each altitude was established to ensure that participants had been adequately exposed to the effects of the respective altitudes.

### 2.3. Measurements

Information was obtained on demographic characteristics, comorbidities, vital signs, physical examination, laboratory parameters, arterial blood gases, diagnostic imaging, evolution of clinical symptoms at admission, and treatment. Shock septic was defined as suspected or confirmed infection, lactate ≥ 2.0 mmol/L, and requirements of vasopressors to maintain a mean arterial pressure of 65 mm Hg or higher after an intravenous fluid load of at least 20 mL/kg over 60 min [24]. Outcome variables included overall mortality, hospital mortality, admission to the ICU, requirement for IMV, and/or vasopressor requirement. In addition, medical records needed to include sufficient information for the evaluation of the CURB-65 score (Supporting table 1).

The retrospective data collection process was conducted by trained healthcare professionals, including physicians and specialized nursing staff. These professionals reviewed the medical records coded with the diagnosis of CAP for patients eligible for admission to the study center. To minimize transcription biases, the data were verified by at least two members of the research team directly from the electronic medical record. This process involved cross-referencing coded data with clinical records and diagnostic documentation to validate the diagnosis of CAP for each case included in the study. In addition, the personnel responsible for data collection received appropriate training to ensure the quality and accuracy of the process.

### 2.4. Sample Size

The sample size was calculated using data from the study by Roca et al. [25], where the ROX index for IMV had a sensitivity of 70.1% and specificity of 72.4% at less than 2500 m above sea level, and data from the study by Reyes et al. [26], where the ROX index had a sensitivity of 77.8% and specificity of 62.4% at an altitude greater than 2500 m above sea level, using the formula for comparing two receiver operating characteristic (ROC) curves in independent samples, with an event incidence of 35.5%, power of 90%, and significance level of 5%, and a minimum of 1913 subjects were required.

### 2.5. Statistical Analysis

Data were directly obtained from electronically obtained medical records, which were reviewed in their entirety, and data collection was performed using the Research Electronic Data Capture (REDCap) electronic data capture software [27]. Subsequently, it was downloaded into an Excel spreadsheet for final analysis using licensed SPSS software. Qualitative variables were summarized in frequency and percentage, quantitative variables in mean and standard deviation (±) if their distribution was normal, and median and interquartile range (IQR) if their distribution was nonnormal; normality tests were performed by the Kolmogorov–Smirnov test. For the analysis of two independent samples, the T-student-Welch test for two samples and the Mann–Whitney *U* test were used [28].

ROC curve were performed, and the area under the ROC curve was calculated for the CURB 65 score, PaO_2_ mmHg ≤ 60, SaO2% ≤ 90, A-a O_2_ gradient ≥ 10, a/A index ≤ 0.8, PaO_2_/FiO_2_ ≤ 300, SpO_2_% ≤ 90, and SpO_2_/FiO_2_ ≤ 350 for outcomes of mortality, IMV, and vasopressor support. To assess the predictive capacity of the CURB-65 score in relation to clinical outcomes, only patients with scores ranging from 2 to 5 were included, excluding those with scores of 0 and 1. This decision was based on evidence showing that patients with lower scores have a significantly lower risk of severe complications, which limits the utility of the CURB-65 score in this group [29]. The calculation of oxygenation parameters, oxygenation indices, and the CURB-65 score was based on clinical information collected during the first 24 h after admission to the emergency department. Sensitivity, specificity, positive predictive value, negative predictive value, positive likelihood ratio (LR+), and negative likelihood ratio (LR–) were calculated. Youden's J statistic was used to determine the optimal cutoff point in the analyzed cohort. A *p* < 0.05 was considered statistically significant.

The calculated areas under ROC curves were compared using the DeLong test [28]. The interpretation of the areas under ROC curves was as follows: 0.50 indicated an absence of discriminatory capacity; 0.51 to 0.60 indicated nearly no discriminatory capacity; 0.61 to 0.69 indicated poor discriminatory capacity; > 0.7 to 0.8 indicated acceptable discriminatory capacity; > 0.80 to 0.90 indicated excellent discriminatory capacity; and > 0.90 indicated outstanding discriminatory capacity [28].

### 2.6. Missing Data

An imputation analysis of missing data was performed for variables with a loss of less than 10%, applying weighted mean imputation for quantitative variables and logistic regression for qualitative variables [28]. Variables with data loss greater than 10% were excluded from the analysis. To ensure that imputation has not biased or altered the study results, a comparison was conducted between nonimputed and imputed results, confirming that there was no difference that significantly modified the original data [28].

## 3. Results

### 3.1. General Characteristics of the Population

**Figure 1:**
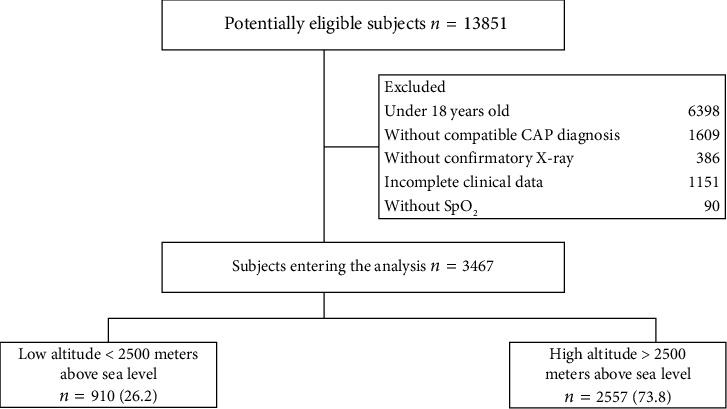
Patient flowchart. Notes: CAP: community-acquired pneumonia; SpO_2_: oxygen saturation by pulse oximetry.

3467 were selected for analysis, with 73.7% (2557/3467) residing at high altitudes and 26.2% (910/3467) at low altitudes (Figure 1). In the total sample, the average age was 67.5 years (±: 20.75), with 59.1% (2050/3467) of them being men (Table 1). The median time from the diagnosis of CAP to death was 6.9 days (IQR: 4–11) in the general population, 5.6 days (IQR: 4–8) in the low altitudes group, and 6.9 days (IQR: 4–12) in the high altitudes group. The low altitude patients had a lower frequency of fever (38.7%, 352/910 vs. 51.9%, 1326/3467) and cyanosis (4.4%, 40/910 vs. 9.4%, 240/3467) compared to the high altitudes resident group. 27.1% (693/3467) of patients at higher altitudes had a history of smoking, compared to 4.2% (38/910) at lower altitudes (*p* < 0.001). Patients at lower altitudes exhibited higher oxygen saturation (89.9, ±: 6.93 vs. 88.5, ±: 7.36, *p* < 0.001).

In patients residing at high altitudes, a significant increase in hemoglobin levels (13.9, ±: 2.1 vs. 12.6, ±: 2.47; *p* < 0.001) and hematocrit (40.2, ±: 7.09 vs. 38.1, ±: 7.64; *p* < 0.001) was observed compared to the group at lower altitudes (Supporting table 2). Conversely, individuals at low altitudes group exhibited higher levels of PO_2_ (69.7, ±: 26.72 vs. 62.5, ±: 17.43; *p* < 0.001), SO_2_ (91.8, ±: 6.34 vs. 88.2, ±: 8.68; *p* < 0.001), and PaO_2_/FiO_2_ ratio (237, ±: 113.26 vs. 228.1, ±: 89.06; *p* < 0.001) compared to the high altitudes group. In contrast, the high altitudes group showed lower values of bicarbonate (20.9, ±: 4.34 vs. 22.9, ±: 6.19; *p* < 0.001), BE (−2.8, ±: 3.98 vs. −1.5, ±: 6.98), and PCO_2_ (33.4, ±: 8.65 vs. 36.4, ±: 12.44; *p* < 0.001).

At high altitudes, 11% (280/2557) of patients required IMV compared to 1.3% (12/910) at low altitudes (*p* < 0.001) (Supporting table 3). Similarly, 10.8% (277/2557) of patients at high altitudes required vasopressor support compared to 1.9% (17/910) of patients at low altitudes (*p* < 0.001). The overall mortality rate was 13.84%, with 14.4% corresponding to patients at high altitudes and 13.3% at low altitudes.

### 3.2. Performance of Oxygenation Parameters, Oxygenation Indices, and CURB-65 at High and Low Altitudes

The CURB-65 score  ≥ 2 showed a performance in predicting mortality of 0.707 (95% CI: 0.653–0.761; *p* < 0.001) at low altitudes and 0.737 (95% CI: 0.709–0.765; *p* < 0.001) at high altitudes (Table 2 and Supporting table 4). The PaO_2_/FiO_2_ ≤ 300 showed a performance in predicting the need for IMV and vasopressor support of 0.734 (95% CI: 0.685–0.783; *p* < 0.001) and 0.724 (0.674–0.775; *p* < 0.001) at high altitudes, respectively. The SpO_2_/FiO_2_ ≤ 350 showed a performance in predicting the need for IMV of 0.679 (95% CI: 0.507–0.85; *p* < 0.001) at low altitudes. The A-a O_2_ gradient ≥ 10 showed a performance in predicting the need for vasopressor support of 0.686 (95% CI: 0.537–0.835; *p*=0.06) at low altitudes and 0.703 (95% CI: 0.662–0.744; *p* < 0.001) at high altitudes.

### 3.3. Comparison of Oxygenation Parameters, Oxygenation Indices, and CURB-65 Performances Between high and Low Altitudes

PaO_2_/FiO_2_ ≤ 300 showed a performance in predicting mortality of 0.567 (95% CI: 0.506–0.627) at low altitudes and 0.667 (95% CI: 0.624–0.709) at high altitudes (*p*=0.008) (Table 3). SpO_2_% ≤ 90 showed a performance in predicting vasopressor support requirement of 0.419 (0.285–0.553) at low altitudes and 0.592 (0.553–0.63) at high altitudes (*p*=0.017). PaO_2_/FiO_2_ ≤ 300 showed a performance in predicting vasopressor support requirement of 0.458 (95% CI: 0.31–0.605) at low altitudes and 0.724 (95% CI: 0.674–0.775) at high altitudes (*p*=0.001).

## 4. Discussion

In this study, we identified a good predictive performance of oxygenation parameters, oxygenation indices, and the CURB-65 score in relation to key outcomes, including mortality, the need for IMV, and vasopressor support. Notably, significant associations were observed with variables such as SpO_2_ ≤ 90%, A-a O_2_ gradient ≥ 10, PaO_2_/FiO_2_ ≤ 300, and SpO_2_/FiO_2_ ≤ 350. It is important to highlight that as altitude increases, there is a corresponding decline in SaO_2_, PaO_2_, and PCO_2_ values, while hemoglobin concentration and hematocrit tend to rise. However, no significant differences in mortality related to altitude were found. The differences observed in the use of vasopressors and mechanical ventilation, despite similar mortality rates, can be attributed to the lower complexity and fewer resources at lower altitudes, resulting in patient transfers to more advanced hospitals. At higher altitudes, although institutions had more experience, the increased severity of patient conditions led to similar mortality rates.

The CURB-65 score exhibited strong predictive performance for mortality in both low- and high-altitudes settings. While its performance was slightly better at high altitudes, the difference was not clinically significant. This reinforces the CURB-65 score's utility as a practical and effective triage tool across various environments [30–32]. In contrast, the performance of other oxygenation indices, such as PaO_2_/FiO_2_ and SpO_2_/FiO_2_, showed notable variations between altitudes, with higher altitudes demonstrating enhanced predictive accuracy for outcomes like the need for IMV and vasopressor support. Nevertheless, these parameters serve as valuable complements to the CURB-65 score, especially in critical cases, by providing additional insights into respiratory status and the severity of hypoxemia [29, 31, 33].

Oxygenation indices have been used to predict the need for IMV and mortality in hospitalized COVID-19 patients. The delta PaO_2_/FiO_2_ ratio demonstrated moderate predictive performance for 28-day IMV (ROC curve: 0.589), 7-day mortality (ROC curve: 0.585), and 28-day mortality (ROC curve: 0.567) [34]. In contrast, PaO_2_/FiO_2_ ≤ 300 exhibited stronger predictive value for IMV (ROC curve: 0.669) [34]. In our study, oxygenation indices like PaO_2_/FiO_2_ ≤ 300 demonstrated stronger performance at high altitudes in predicting the need for IMV and vasopressor support. In addition, SpO_2_/FiO_2_ ≤ 350 and the A-a O_2_ gradient ≥ 10 provided valuable predictive information, with SpO_2_/FiO_2_ showing good performance for predicting IMV at low altitudes. The observed differences in performance between high and low altitudes indicate that while oxygenation parameters such as PaO_2_/FiO_2_ are more predictive at higher altitudes, the CURB-65 score remains a reliable tool in both settings [18, 29, 33]. These findings underscore the importance of incorporating both oxygenation indices and clinical scores, particularly in critical care environments at varying altitudes [35, 36].

Simbaña-Rivera et al. [8] described clinical outcomes in patients with IMV admitted to the ICU due to viral pneumonia, dividing them into two groups according to altitude. Findings revealed that patients at higher altitudes had a higher likelihood of survival and a shorter stay in the ICU. Although these results differ from ours, it is important to consider factors such as the complexity level of medical centers and the presence of an older population with comorbidities, which may have contributed to a higher incidence of septic shock. This, in turn, could explain a higher frequency of IMV and vasopressor support, along with a prolonged stay in the ICU [37, 38].

We selected the CURB-65 score for its practicality and ease of use in our hospital setting, even though we also considered the sequential organ failure assessment (SOFA) and PSI scores [30–33]. While SOFA could provide additional insights, the CURB-65 score was more appropriate for this study. Future research will include a detailed evaluation of the SOFA and PSI scores. In addition, further studies are needed to better characterize patients' conditions at various altitudes and examine their relationships with oxygenation indices, demographic factors, and laboratory tests as clinical predictors [30–34]. This research is crucial for guiding treatment and minimizing complications associated with CAP.

In our study, we identified potential confounding variables through a thorough review of the literature to detect those commonly recognized as confounders [28]. In addition, we consulted with medical experts, including pulmonologists and intensivists, to identify variables that could influence the relationship between exposure and outcome. We conducted a bivariate analysis to explore the association between exposure and outcome. Finally, we validated our findings by including variables from previous studies and similar scenarios, as well as using external datasets to ensure that the observed effects are not specific to our sample [28].

### 4.1. Limitations

Our study has some limitations, such as the retrospective collection of data from medical records. However, the research team has solid experience in the interpretation, extraction, and appropriate synthesis of this type of information. The inclusion of a small number of patients at low altitudes limits the generalization of results to populations with similar characteristics. Despite being a retrospective study based on medical records, measures were implemented to minimize information bias, such as the training of the personnel in charge of collecting medical data and the construction of the manuscript based on the checklist of items that should be included in the cohort study reports (Supporting table 5).

We excluded individuals with survival of less than 30 days to ensure that our study focused on patients who had a sufficient duration to observe relevant clinical outcomes and treatment effects. This decision was made to reduce potential confounding that could arise from very short-term survival cases. However, we recognize that excluding these patients could introduce bias toward less severe cases.

In our study, the initial sample size calculation was based on an expected incidence rate of 35% for the need for IMV [25, 26]. However, the observed data revealed a considerably lower IMV rate of 11%. This discrepancy may impact the statistical power of the study in several ways [28]. First, the originally calculated sample size may be larger than necessary to detect effects at the observed incidence rate, potentially leading to an increased ability to identify smaller differences between groups [28]. However, a larger sample size does not always guarantee better statistical power if the actual incidence is much lower than anticipated. In addition, with a lower IMV rate, the study's capacity to detect significant associations may be diminished, as variability in event rates can increase the risk of Type II error (failing to detect a true effect), especially in analyses that do not adequately account for the discrepancy in incidence [28].

## 5. Conclusion

In patients with CAP at altitudes above 2500 m above sea level, both the oxygenation parameters, oxygenation indices, and the CURB-65 score demonstrate improved predictive capability in terms of mortality, need for IMV, and requirement for vasopressor support. These findings are attributed to the specific conditions and compensatory mechanisms experienced by these patients, highlighting the crucial importance of considering altitude in the management and treatment of pneumonia in this context.

## Figures and Tables

**Table 1:** General characteristics of the population.

	Total population *n* = 3467	High altitudes *n* = 2557	Low altitudes *n* = 910	*p*-value
Age (years), mean (±)	67.5 (20.75)	67.4 (21.18)	67.8 (19.49)	0.616
Male, *n* (%)	2050 (59.1)	1540 (60.2)	510 (56)	0.028
Hypertension, *n* (%)	1779 (51.3)	1414 (55.3)	365 (40.1)	< 0.001
Smoking, *n* (%)	731 (21.1)	693 (27.1)	38 (4.2)	< 0.001
Chronic heart failure, *n* (%)	581 (16.8)	425 (16.6)	156 (17.1)	0.717
Chronic lung disease, *n* (%)	1024 (29.5)	727 (28.4)	297 (32.6)	0.017
Cough, *n* (%)	2839 (81.9)	2116 (82.8)	723 (79.5)	0.026
Dyspnea, *n* (%)	2486 (71.7)	1815 (71)	671 (73.7)	0.113
Fever, *n* (%)	1678 (48.4)	1326 (51.9)	352 (38.7)	< 0.001
Cyanosis, *n* (%)	280 (8.1)	240 (9.4)	40 (4.4)	< 0.001
Heart rate, mean (±)	91 (18.74)	91.8 (19.18)	88.6 (17.23)	< 0.001
Mean arterial pressure, mean (±)	88.7 (16.73)	88.5 (14.99)	89.2 (20.85)	0.334
Temperature, mean (±)	36.8 (1.36)	36.9 (1.5)	36.7 (0.9)	< 0.001
Altered Glasgow, *n* (%)	236 (12.7)	227 (12.7)	9 (11.5)	< 0.001
Saturation, mean (±)	88.9 (7.27)	88.5 (7.36)	89.9 (6.93)	< 0.001
FiO2 intake, mean (±)	26.3 (10.35)	26.2 (9.93)	26.5 (11.44)	0.549

*Notes:* ±: standard deviation; *n*: number; FiO_2_: inspired fraction of oxygen.

**Table 2:** Predictive capability of oxygenation indices and the CURB-65 score at low and high altitudes.

	ROC curve (95% IC)	
	Low altitudes *n* = 910	High altitudes *n* = 2557
*Mortality*		
CURB 65 ≥ 2	0.707 (0.653–0.761)	0.737 (0.709–0.765)
PaO_2_ mmHg ≤ 60	0.495 (0.433–0.556)	0.468 (0.427–0.509)
SaO_2_% ≤ 90	0.401 (0.208–0.593)	0.547 (0.5–0.594)
A-a O_2_ gradient ≥ 10	0.628 (0.509–0.747)	0.668 (0.633–0.704)
a/A index ≤ 0.8	0.603 (0.486–0.719)	0.656 (0.62–0.692)
PaO2/FiO2 ≤ 300	0.567 (0.506–0.627)	0.667 (0.624–0.709)
SpO_2_% ≤ 90	0.56 (0.495–0.625)	0.58 (0.545–0.614)
SpO_2_/FiO_2_ ≤ 350	0.686 (0.63–0.742)	0.687 (0.657–0.718)

*Invasive mechanical ventilation*		
CURB 65 ≥ 2	0.500 (0.333–0.667)	0.573 (0.536–0.61)
PaO_2_ mmHg ≤ 60	0.624 (0.455–0.793)	0.481 (0.431–0.53)
SaO_2_% ≤ 90	0.330 (0.000–0.695)	0.567 (0.517–0.617)
A-a O_2_ gradient ≥ 10	0.671 (0.500–0.842)	0.724 (0.684–0.764)
a/A index ≤ 0.8	0.647 (0.47–0.824)	0.719 (0.68–0.758)
PaO_2_/FiO_2_ ≤ 300	0.662 (0.466–0.858)	0.734 (0.685–0.783)
SpO_2_% ≤ 90	0.426 (0.234–0.619)	0.614 (0.574–0.653)
SpO_2_/FiO_2_ ≤ 350	0.679 (0.507–0.85)	0.691 (0.652–0.729)

*Vasopressor support*		
CURB 65 ≥ 2	0.672 (0.527–0.817)	0.604 (0.569–0.64)
PaO_2_ mmHg ≤ 60	0.663 (0.52–0.807)	0.478 (0.429–0.527)
SaO_2_% ≤ 90	0.553 (0.359–0.746)	0.573 (0.523–0.622)
A-a O_2_ gradient ≥ 10	0.686 (0.537–0.835)	0.703 (0.662–0.744)
a/A index ≤ 0.8	0.685 (0.54–0.831)	0.697 (0.657–0.738)
PaO_2_/FiO_2_ ≤ 300	0.458 (0.31–0.605)	0.724 (0.674–0.775)
SpO_2_% ≤ 90	0.419 (0.285–0.553)	0.592 (0.553–0.63)
SpO_2_/FiO_2_ ≤ 350	0.566 (0.419–0.713)	0.668 (0.629–0.708)

*Note:* PaO_2_: arterial oxygen pressure, SaO_2_: arterial oxygen saturation, PaO_2_/FiO_2_ ratio: arterial oxygen pressure/inspired fraction of oxygen, SpO_2_: oxygen saturation by pulse oximetry, SaO_2_/FiO_2_ ratio: oxygen saturation by pulse oximetry in relation to the inspired oxygen fraction.

Abbreviations: CI, confidence interval; ROC curve, receiver operating characteristic curve.

**Table 3:** Predictive capability of oxygenation indices and the CURB-65 score between low and high altitudes.

	ROC curve (95% IC)		
	Low altitudes *n* = 910	High altitudes *n* = 2557	*p*-value⁣^∗^
*Mortality*			
CURB 65 ≥ 2	0.707 (0.653–0.761)	0.737 (0.709–0.765)	0.312
PaO_2_ mmHg ≤ 60	0.495 (0.433–0.556)	0.468 (0.427–0.509)	0.476
SaO_2_% ≤ 90	0.401 (0.208–0.593)	0.547 (0.5–0.594)	0.167
A-a O_2_ gradient ≥ 10	0.628 (0.509–0.747)	0.668 (0.633–0.704)	0.527
a/A index ≤ 0.8	0.603 (0.486–0.719)	0.656 (0.62–0.692)	0.398
PaO_2_/FiO_2_ ≤ 300	0.567 (0.506–0.627)	0.667 (0.624–0.709)	0.008
SpO_2_% ≤ 90	0.56 (0.495–0.625)	0.58 (0.545–0.614)	0.601
SpO_2_/FiO_2_ ≤ 350	0.686 (0.63–0.742)	0.687 (0.657–0.718)	0.962

*Invasive mechanical ventilation*			
CURB 65 ≥ 2	0.5 (0.333–0.667)	0.573 (0.536–0.61)	0.405
PaO_2_ mmHg ≤ 60	0.624 (0.455–0.793)	0.481 (0.431–0.53)	0.125
SaO2% ≤ 90	0.33 (0–0.695)	0.567 (0.517–0.617)	0.273
A-a O_2_ gradient ≥ 10	0.671 (0.5–0.842)	0.724 (0.684–0.764)	0.576
a/A index ≤ 0.8	0.647 (0.47–0.824)	0.719 (0.68–0.758)	0.462
PaO_2_/FiO_2_ ≤ 300	0.662 (0.466–0.858)	0.734 (0.685–0.783)	0.503
SpO_2_% ≤ 90	0.426 (0.234–0.619)	0.614 (0.574–0.653)	0.072
SpO_2_/FiO_2_ ≤ 350	0.679 (0.507–0.85)	0.691 (0.652–0.729)	0.899

*Vasopressor support*			
CURB 65 ≥ 2	0.672 (0.527–0.817)	0.604 (0.569–0.64)	0.377
PaO_2_ mmHg ≤ 60	0.663 (0.52–0.807)	0.478 (0.429–0.527)	0.019
SaO_2_% ≤ 90	0.553 (0.359–0.746)	0.573 (0.523–0.622)	0.853
A-a O_2_ gradient ≥ 10	0.686 (0.537–0.835)	0.703 (0.662–0.744)	0.836
a/A index ≤ 0.8	0.685 (0.54–0.831)	0.697 (0.657–0.738)	0.880
PaO2/FiO_2_ ≤ 300	0.458 (0.31–0.605)	0.724 (0.674–0.775)	0.001
SpO_2_% ≤ 90	0.419 (0.285–0.553)	0.592 (0.553–0.63)	0.017
SpO_2_/FiO_2_ ≤ 350	0.566 (0.419–0.713)	0.668 (0.629–0.708)	0.200

*Note:* PaO_2_: arterial oxygen pressure, SaO_2_: arterial oxygen saturation, PaO_2_/FiO_2_ ratio: arterial oxygen pressure/inspired fraction of oxygen, SpO_2_: oxygen saturation by pulse oximetry, SaO_2_/FiO_2_ ratio: oxygen saturation by pulse oximetry in relation to the inspired oxygen fraction.

Abbreviations: CI, confidence interval; ROC curve, receiver operating characteristic curve.

⁣^∗^DeLong test.

## Data Availability

The data supporting the findings of this study are not publicly available; they will only be shared by the corresponding author upon reasonable request and at the authors' discretion.

## Supporting Information

Supporting Table 1: Risk scores used in the study. Notes: ROC curve: receiver operating characteristic curve.

Supporting Table 2: Laboratory tests. Notes: ±: standard deviation, PaO_2_: arterial oxygen pressure, PaCO_2_: arterial carbon dioxide pressure, HCO_3_: bicarbonate, BE: base excess, SaO_2_: arterial oxygen saturation, FiO_2_: inspired fraction of oxygen, PaO_2_/FiO_2_ ratio: arterial oxygen pressure/inspired fraction of oxygen.

Supporting table 3: Medical treatment and complications. Notes: *n*: number, m: media, SD: standard deviation, ICU: intensive care unit, IMV: invasive mechanical ventilation, NIMV: noninvasive mechanical ventilation.

^a^Suspected or confirmed infection, plus lactate ≥ 2.0 mmol/L, and requirements of vasopressors to maintain a mean arterial pressure of 65 mm·Hg or higher after an intravenous fluid load of at least 20 mL/kg over 60 min.

⁣^∗^12-month mortality.

Supporting table 4: Performance of oxygenation indices and the CURB-65 score at low and high altitudes. Notes: SE: sensitivity; SP: specificity; PPV: positive predictive value; NPV: negative predictive value; LR: likelihood ratio.

Supporting table 5: STROBE statement—checklist of items that should be included in reports of cohort studies. ⁣^∗^Give information separately for exposed and unexposed groups. N/A: not applicable.

Note: An explanation and elaboration article discusses each checklist item and gives methodological background and published examples of transparent reporting. The STROBE checklist is best used in conjunction with this article (freely available on the Web sites of PLoS Medicine at https://www.plosmedicine.org/, Annals of Internal Medicine at https://www.annals.org/, and Epidemiology at https://www.epidem.com/). Information on the STROBE initiative is available at https://www.strobe-statement.org.
