# Supplementary material for: Comparison of the Predictive Capacity of Oxygenation Parameters, Oxygenation Indices, and CURB-65 to Mortality, Mechanical Ventilation, and Vasopressor Support in Community-Acquired Pneumonia at Different Altitudes
Source: Can Respir J. 2025 Mar 11;2025:9378618. doi: 10.1155/carj/9378618 (PMC11991769; doi:10.1155/carj/9378618)
Supplement: Supporting Information — Additional supporting information can be found online in the Supporting Information section. [file 9378618.f1.pdf]

Supporting Table 1. Risk scores used in the study.

CURB 65	Points	Classification	Management	Cohort points ROC-curve
**C**onfusion<br>**U**remia >19 mg/ml<br>**R**espiratory rate >30 rpm<br>**B**lood pressure: Systolic blood pressure < 90 mmHg or dyastolic < 60 mmHg<br>≥ **65** years	0-1	Low risk	Outpatient treatment	
	2	Moderate risk	Hospital admission versus observation admission	≥2
	>/3	High risk	Inpatient treatment	

Notes: ROC-curve: receiver operating characteristic curve.

Supporting table 2. Laboratory tests

	Total population n= 3467	High altitud n= 2557	Low altitud n= 910	p-value
Leukocytes x10^9 / L, mean (+/-)	12.6 (8.1)	12.6 (8.4)	12.6 (6.9)	0.862
Hemoglobin g/dl, mean (+/-)	13.5 (19.11)	13.9 (22.1)	12.6 (2.47)	0.005
Hematocrit %, mean (+/-)	39.7 (7.29)	40.2 (7.09)	38.1 (7.64)	<0.001
Platelets x10^9 / L, mean (+/-)	259 (119)	256 (117)	269 (123)	0.005
Creatinine, mean (+/-)	1.3 (2.45)	1.3 (2.61)	1.5 (1.46)	0.003
blood urea nitrogen, mean (+/-)	25.7 (19.54)	25 (18.35)	29.8 (25.54)	<0.001
pH, mean (+/-)	7.41 (0.07)	7.41 (0.07)	7.41 (0.1)	0.902
PaO2, mean (+/-)	65.4 (21.95)	62.5 (17.43)	69.7 (26.72)	<0.001
PaCO2, mean (+/-)	33.7 (9.16)	33.4 (8.65)	36.4 (12.44)	<0.001
HCO3, mean (+/-)	21.1 (4.59)	20.9 (4.34)	22.9 (6.19)	<0.001
BE, mean (+/-)	-2.6 (4.32)	-2.8 (3.98)	-1.5 (6.98)	<0.001
Lactate, mean (+/-)	6.8 (84.48)	7.4 (89.62)	2.4 (2.05)	0.005
SaO2, mean (+/-)	88.5 (8.59)	88.2 (8.68)	91.8 (6.34)	<0.001
FiO2, mean (+/-)	29.8 (13.24)	29.2 (12.29)	36 (19.45)	<0.001
PaO2/FiO2, mean (+/-)	232.1 (100.68)	228.1 (89.06)	237 (113.26)	0.031

Notes: +/-: standard deviation, PaO2: arterial oxygen pressure, PaCO2: arterial carbon dioxide pressure, HCO3: bicarbonate, BE: base excess, SaO2: arterial oxygen saturation, FiO2: inspired fraction of oxygen, PaO2/FiO2 ratio: arterial oxygen pressure/inspired fraction of oxygen.

Supporting table 3. Medical treatment and complications.

	Total population n= 3467	High altitud n= 2557	Low altitud n= 910	p-value
[a]Septic shock, n(%)	369 (10.6)	335 (13.1)	34 (3.7)	<0.001
Vasopressor support, n(%)	294 (8.5)	277 (10.8)	17 (1.9)	<0.001
Use of corticosteroid, n(%)	831 (24)	571 (22.3)	260 (28.6)	<0.001
ICU requirement, n(%)	430 (12.4)	400 (15.6)	30 (3.3)	<0.001
Days of stay in ICU, mean (+/-)	9.9 (12.12)	10 (12.25)	6.7 (6.13)	<0.001
IMV, n(%)	292 (8.4)	280 (11)	12 (1.3)	<0.001
NIMV, n(%)	187 (5.4)	120 (4.7)	67 (7.4)	0.002
Hospitalization requirement, n(%)	3185 (91.9)	2313 (90.5)	872 (95.9)	<0.001
Days of stay in Hospitalization, mean (+/-)	10.6 (66.85)	12.1 (77.36)	6.2 (13.89)	<0.001
*Mortality, n(%)	480 (13.84)	359 (14.04)	121 (13.3)	0.577

Notes: n: number, m: media, SD: Standard deviation, ICU: intensive care unit, IMV: invasive mechanical ventilation, NIMV: Non-invasive mechanical ventilation.

[a] Suspected or confirmed infection, plus lactate ≥2.0 mmol/L and requirements of vasopressors to maintain a mean arterial pressure of 65 mm Hg or higher after an intravenous fluid load of at least 20 mL/kg over 60 minutes.

*12-month mortality.

Supporting table 4. Performance of oxygenation indices and CURB-65 at low and high altitude.

	S (CI 95%)	E (CI 95%)	VPP (CI 95%)	VPN (CI 95%)	LR+ (CI 95%)	LR- (CI 95%)
**Mortality- Low altitude**						
CURB 65 ≥ 2	79.3 (76.7-82)	50.2 (47-53.5)	15.2 (12.9-17.5)	95.6 (94.2-96.9)	1.59 (1.407-1.807)	0.41 (0.363-0.411)
PaO2 mmHg ≤ 60	60.9 (57.7-64)	35.2 (32.1-38.3)	9.6 (7.6-11.5)	88.9 (86.8-90.9)	0.94 (0.791-1.115)	1.11 (0.936-1.319)
SaO2% ≤90	88.9 (82.5-95.3)	21.4 (13.1-29.8)	10.8 (4.5-17.1)	94.7 (90.2-99.3)	1.13 (0.875-1.462)	0.52 (0.401-0.67)
A-a O_2 Gradient ≥10	87 (82.1-91.8)	32.7 (26-39.4)	15.3 (10.1-20.4)	94.7 (91.5-97.9)	1.29 (1.068-1.564)	0.4 (0.329-0.482)
a/A index ≤0.8	17.4 (12-22.8)	61.8 (54.9-68.8)	6 (2.6-9.4)	84.3 (79.1-89.5)	0.46 (0.183-1.133)	1.34 (0.537-3.325)
PaO2/FiO2 ≤300	16.3 (13.9-18.7)	75.8 (73-78.6)	7 (5.4-8.7)	89 (86.9-91)	0.67 (0.417-1.087)	1.1 (0.684-1.782)
SpO2% ≤90	58.7 (55.5-61.9)	34.5 (31.4-37.6)	9.2 (7.3-11)	88.1 (86-90.2)	0.9 (0.749-1.071)	1.2 (1.002-1.432)
SpO2/FiO2 ≤350	39.1 (36-42.3)	31.1 (28-34.1)	6 (4.5-7.5)	81.9 (79.4-84.4)	0.57 (0.438-0.735)	1.96 (1.513-2.54)
**Invasive mechanical ventilation- Low altitude**						
CURB 65 ≥ 2	41.7 (38.5-44.9)	47.1 (43.9-50.3)	1 (0.4-1.7)	98.4 (97.5-99.2)	0.79 (0.402-1.543)	1.24 (0.632-2.426)
PaO2 mmHg ≤ 60	66.7 (63.6-69.7)	35.6 (32.5-38.7)	1.4 (0.6-2.1)	98.8 (98-99.5)	1.04 (0.692-1.55)	0.94 (0.625-1.4)
SaO2% ≤90	75 (66.2-83.8)	20.2 (12.1-28.4)	4.1 (0-8.1)	94.7 (90.2-99.3)	0.94 (0.529-1.671)	1.24 (0.695-2.198)
A-a O_2 Gradient ≥10	87.5 (82.8-92.2)	31.1 (24.5-37.7)	5.3 (2.1-8.6)	98.2 (96.4-100)	1.27 (0.96-1.68)	0.4 (0.304-0.531)
a/A index ≤0.8	12.5 (7.8-17.2)	62.8 (55.9-69.7)	1.5 (-0.3-3.2)	94.2 (90.8-97.5)	0.34 (0.053-2.121)	1.39 (0.221-8.804)
PaO2/FiO2 ≤300	16.7 (14.2-19.1)	76.5 (73.7-79.3)	0.9 (0.3-1.6)	98.6 (97.8-99.3)	0.71 (0.199-2.527)	1.09 (0.306-3.881)
SpO2% ≤90	58.3 (55.1-61.5)	35.1 (32-38.2)	1.2 (0.5-1.9)	98.4 (97.6-99.2)	0.9 (0.556-1.453)	1.19 (0.735-1.921)
SpO2/FiO2 ≤350	41.7 (38.5-44.9)	33.7 (30.7-36.8)	0.8 (0.2-1.4)	97.7 (96.8-98.7)	0.63 (0.321-1.23)	1.73 (0.884-3.382)
**Vasopressor support- Low altitude**						
CURB 65 ≥ 2	76.5 (73.7-79.2)	47.7 (44.5-50.9)	2.7 (1.7-3.8)	99.1 (98.4-99.7)	1.46 (1.115-1.917)	0.49 (0.376-0.647)
PaO2 mmHg ≤ 60	70.6 (67.6-73.5)	35.7 (32.6-38.8)	2 (1.1-3)	98.5 (97.7-99.3)	1.1 (0.805-1.498)	0.82 (0.603-1.123)
SaO2% ≤90	81.8 (74-89.7)	20.7 (12.5-29)	12.2 (5.5-18.8)	89.5 (83.2-95.7)	1.03 (0.765-1.393)	0.88 (0.65-1.184)
A-a O_2 Gradient ≥10	88.9 (84.4-93.4)	31.3 (24.7-37.9)	6.1 (2.7-9.5)	98.2 (96.4-100.1)	1.29 (1.006-1.663)	0.36 (0.276-0.457)
a/A index ≤0.8	11.1 (6.6-15.6)	62 (55.1-68.9)	1.4 (-0.3-3.2)	93.3 (89.7-96.9)	0.29 (0.046-1.874)	1.43 (0.224-9.184)
PaO2/FiO2 ≤300	29.4 (26.5-32.4)	76.7 (74-79.5)	2.3 (1.4-3.3)	98.3 (97.4-99.1)	1.26 (0.599-2.663)	0.92 (0.436-1.94)
SpO2% ≤90	70.6 (67.6-73.5)	35.3 (32.2-38.4)	2 (1.1-3)	98.4 (97.6-99.2)	1.09 (0.799-1.488)	0.83 (0.611-1.138)
SpO2/FiO2 ≤350	58.8 (55.6-62)	33.9 (30.9-37)	1.7 (0.8-2.5)	97.7 (96.8-98.7)	0.89 (0.597-1.329)	1.21 (0.813-1.811)
**Mortality- High altitude**						
CURB 65 ≥ 2	60.7 (58.8-62.6)	48.4 (46.4-50.3)	12.6 (11.3-13.9)	90.9 (89.8-92)	1.18 (1.061-1.302)	0.81 (0.733-0.9)
PaO2 mmHg ≤ 60	48.7 (46-51.4)	50.2 (47.5-52.9)	11.4 (9.7-13.1)	88.2 (86.4-89.9)	0.98 (0.825-1.161)	1.02 (0.861-1.212)
SaO2% ≤90	46.2 (43.5-48.8)	44.9 (42.2-47.5)	11.3 (9.6-12.9)	84.6 (82.7-86.5)	0.84 (0.71-0.987)	1.2 (1.017-1.415)
A-a O_2 Gradient ≥10	89.2 (87.9-90.6)	17 (15.4-18.7)	13.3 (11.9-14.8)	91.7 (90.5-92.9)	1.08 (1.026-1.129)	0.63 (0.602-0.662)
a/A index ≤0.8	12.7 (11.3-14.2)	74.1 (72.1-76)	6.6 (5.5-7.7)	85.6 (84-87.1)	0.49 (0.352-0.686)	1.18 (0.844-1.644)
PaO2/FiO2 ≤300	8.3 (6.7-9.9)	83.3 (81.1-85.4)	6.8 (5.3-8.3)	86 (84-88)	0.49 (0.283-0.865)	1.1 (0.629-1.928)
SpO2% ≤90	43.6 (41.6-45.5)	38.9 (37-40.8)	8.1 (7-9.1)	84.9 (83.5-86.3)	0.71 (0.622-0.818)	1.45 (1.264-1.664)
SpO2/FiO2 ≤350	36.4 (34.6-38.3)	43.9 (42-45.8)	7.4 (6.4-8.4)	84.9 (83.5-86.3)	0.65 (0.554-0.761)	1.45 (1.235-1.697)
**Invasive mechanical ventilation- High altitude**						
CURB 65 ≥ 2	64.6 (62.8-66.5)	48.8 (46.9-50.8)	13.3 (12-14.6)	91.9 (90.9-93)	1.26 (1.147-1.39)	0.72 (0.658-0.798)
PaO2 mmHg ≤ 60	48.3 (45.7-51)	50.2 (47.5-52.8)	10.9 (9.3-12.6)	88.5 (86.8-90.2)	0.97 (0.815-1.155)	1.03 (0.865-1.226)
SaO2% ≤90	44.8 (42.2-47.4)	44.7 (42-47.3)	11 (9.4-12.7)	84.1 (82.2-86)	0.81 (0.684-0.958)	1.24 (1.044-1.463)
A-a O_2 Gradient ≥10	89.3 (88-90.7)	17 (15.4-18.7)	13 (11.5-14.5)	92 (90.8-93.2)	1.08 (1.026-1.13)	0.63 (0.596-0.656)
a/A index ≤0.8	14.8 (13.2-16.3)	74.4 (72.5-76.3)	7.4 (6.2-8.5)	86.3 (84.8-87.8)	0.58 (0.422-0.787)	1.15 (0.839-1.565)
PaO2/FiO2 ≤300	9.9 (8.1-11.6)	83.5 (81.4-85.7)	8 (6.4-9.5)	86.5 (84.5-88.5)	0.6 (0.357-1.003)	1.08 (0.644-1.81)
SpO2% ≤90	46.9 (45-48.9)	39.3 (37.4-41.2)	8.6 (7.5-9.7)	85.9 (84.6-87.3)	0.77 (0.68-0.881)	1.35 (1.185-1.535)
SpO2/FiO2 ≤350	36.8 (35-38.7)	44 (42.1-45.9)	1.2 (0.8-1.7)	85.1 (83.8-86.5)	0.66 (0.561-0.77)	1.44 (1.226-1.683)
**Vasopressor support- High altitude**						
CURB 65 ≥ 2	64.6 (62.8-66.5)	48.8 (46.9-50.8)	13.3 (12-14.6)	91.9 (90.9-93)	1.26 (1.147-1.39)	0.72 (0.658-0.798)
PaO2 mmHg ≤ 60	48.3 (45.7-51)	50.2 (47.5-52.8)	10.9 (9.3-12.6)	88.5 (86.8-90.2)	0.97 (0.815-1.155)	1.03 (0.865-1.226)
SaO2% ≤90	44.8 (42.2-47.4)	44.7 (42-47.3)	11 (9.4-12.7)	84.1 (82.2-86)	0.81 (0.684-0.958)	1.24 (1.044-1.463)
A-a O_2 Gradient ≥10	89.3 (88-90.7)	17 (15.4-18.7)	13 (11.5-14.5)	92 (90.8-93.2)	1.08 (1.026-1.13)	0.63 (0.596-0.656)
a/A index ≤0.8	14.8 (13.2-16.3)	74.4 (72.5-76.3)	7.4 (6.2-8.5)	86.3 (84.8-87.8)	0.58 (0.422-0.787)	1.15 (0.839-1.565)
PaO2/FiO2 ≤300	9.9 (8.1-11.6)	83.5 (81.4-85.7)	8 (6.4-9.5)	86.5 (84.5-88.5)	0.6 (0.357-1.003)	1.08 (0.644-1.81)
SpO2% ≤90	46.9 (45-48.9)	39.3 (37.4-41.2)	8.6 (7.5-9.7)	85.9 (84.6-87.3)	0.77 (0.68-0.881)	1.35 (1.185-1.535)
SpO2/FiO2 ≤350	36.8 (35-38.7)	44 (42.1-45.9)	1.2 (0.8-1.7)	85.1 (83.8-86.5)	0.66 (0.561-0.77)	1.44 (1.226-1.683)

Notes: SE: sensitivity; SP: specificity; PPV: positive predictive value; NPV: negative predictive value; LR: like hood ratio.

Supporting table 5. STROBE Statement—Checklist of items that should be included in reports of ***cohort studies***

	Item No	Recommendation	Page No
**Title and abstract**	1	(*a*) Indicate the study's design with a commonly used term in the title or the abstract<br>(*b*) Provide in the abstract an informative and balanced summary of what was done and what was found	1
**Introduction**			
Background/rationale	2	Explain the scientific background and rationale for the investigation being reported	2,3
Objectives	3	State specific objectives, including any prespecified hypotheses	2,3
**Methods**			
Study design	4	Present key elements of study design early in the paper	4
Setting	5	Describe the setting, locations, and relevant dates, including periods of recruitment, exposure, follow-up, and data collection	4
Participants	6	(*a*) Give the eligibility criteria, and the sources and methods of selection of participants. Describe methods of follow-up<br>(*b*) For matched studies, give matching criteria and number of exposed and unexposed	4
Variables	7	Clearly define all outcomes, exposures, predictors, potential confounders, and effect modifiers. Give diagnostic criteria, if applicable	4
Data sources/ measurement	8*	For each variable of interest, give sources of data and details of methods of assessment (measurement). Describe comparability of assessment methods if there is more than one group	4,5
Bias	9	Describe any efforts to address potential sources of bias	5
Study size	10	Explain how the study size was arrived at	5
Quantitative variables	11	Explain how quantitative variables were handled in the analyses. If applicable, describe which groupings were chosen and why	5
Statistical methods	12	(*a*) Describe all statistical methods, including those used to control for confounding<br>(*b*) Describe any methods used to examine subgroups and interactions<br>(*c*) Explain how missing data were addressed<br>(*d*) If applicable, explain how loss to follow-up was addressed<br>(*e*) Describe any sensitivity analyses	5,6
**Results**			
Participants	13*	(a) Report numbers of individuals at each stage of study—eg numbers potentially eligible, examined for eligibility, confirmed eligible, included in the study, completing follow-up, and analysed<br>(b) Give reasons for non-participation at each stage<br>(c) Consider use of a flow diagram	7
Descriptive data	14*	(a) Give characteristics of study participants (eg demographic, clinical, social) and information on exposures and potential confounders<br>(b) Indicate number of participants with missing data for each variable of interest<br>(c) Summarise follow-up time (eg, average and total amount)	7
Outcome data	15*	Report numbers of outcome events or summary measures over time	7

Main results	16	(*a*) Give unadjusted estimates and, if applicable, confounder-adjusted estimates and their precision (eg, 95% confidence interval). Make clear which confounders were adjusted for and why they were included<br>(*b*) Report category boundaries when continuous variables were categorized<br>(*c*) If relevant, consider translating estimates of relative risk into absolute risk for a meaningful time period	N/A
Other analyses	17	Report other analyses done—eg analyses of subgroups and interactions, and sensitivity analyses	7
**Discussion**			
Key results	18	Summarise key results with reference to study objectives	8
Limitations	19	Discuss limitations of the study, taking into account sources of potential bias or imprecision. Discuss both direction and magnitude of any potential bias	8-10
Interpretation	20	Give a cautious overall interpretation of results considering objectives, limitations, multiplicity of analyses, results from similar studies, and other relevant evidence	8-10
Generalisability	21	Discuss the generalisability (external validity) of the study results	9,10
**Other information**			
Funding	22	Give the source of funding and the role of the funders for the present study and, if applicable, for the original study on which the present article is based	11

*Give information separately for exposed and unexposed groups. N/A: Not applicable.

Note: An Explanation and Elaboration article discusses each checklist item and gives methodological background and published examples of transparent reporting. The STROBE checklist is best used in conjunction with this article (freely available on the Web sites of PLoS Medicine at http://www.plosmedicine.org/, Annals of Internal Medicine at http://www.annals.org/, and Epidemiology at http://www.epidem.com/). Information on the STROBE Initiative is available at http://www.strobe-statement.org.
